# The influence of recipe disaggregation in dietary assessment: results from the national food consumption survey in Saint Kitts and Nevis

**DOI:** 10.3389/fnut.2024.1404932

**Published:** 2024-09-05

**Authors:** Sandra Patricia Crispim, Vanessa Cardozo Mendes Elias, Latoya Matthew-Duncan, Isabella Francis-Grandeson, Fransen Jean, Victoria Padula de Quadros, Agnieszka Balcerzak, Rita Ferreira de Sousa, Ariel de Moraes Frauches, Claudia Choma Bettega Almeida, Sharon D. Hutchinson, U. Ruth Charrondière, Bridget Anna Holmes

**Affiliations:** ^1^Postgraduate Program in Food and Nutrition, Federal University of Paraná, Curitiba, Paraná, Brazil; ^2^Health Promotion Unit, Ministry of Health, Basseterre, Saint Kitts and Nevis; ^3^Department of Agricultural Economics and Extension, The University of the West Indies, St. Augustine, Trinidad and Tobago; ^4^Food and Agriculture Organization of the United Nations (FAO), Bridgetown, Barbados; ^5^Food and Agriculture Organization of the United Nations (FAO), Rome, Italy; ^6^Formerly of Food and Agriculture Organization of the United Nations (FAO), Rome, Italy

**Keywords:** recipes, food consumption, dietary survey, 24-hour dietary recall, caribbean

## Abstract

It is not always the case that recipe disaggregation is performed in dietary surveys. This investigation aimed to assess the influence of recipe disaggregation in the 2020–2021 national dietary survey in Saint Kitts and Nevis, and provide recommendations for future assessments. A total of 1,004 individuals provided information on their food consumption obtained using 24-h dietary recalls, and 442 recipes were reported. Some recipes were reported as single ingredients at the data collection stage (*n* = 65). In most cases, the respondent provided a standard recipe without disaggregation (*n* = 377). A simple and pragmatic recipe disaggregation methodology was developed. The procedure of recipe disaggregation comprised nine steps, including identifying recipes, ingredients, quantities, conversion factors, and the presence of visible fluid, among others. Seventy-eight non-disaggregated standard recipes were post-disaggregated (21% of recipes) to identify ingredient weights. Either the chi-square or Fisher’s exact tests were applied to assess the significance of differences in frequency of food group consumption before and after disaggregation. The proportion of consumers across the different food groups increased dramatically for some food groups after recipe disaggregation, with significant differences (all *p* < 0.01) for cereals and their products (81.3% before and 94.7% after), eggs and their products (21.7% before and 34.6% after), fats and oils (6.9% before and 44.5% after), fish, shellfish and their products (26.7% before and 38.5% after), meat and meat products (59.7% before and 71.4% after), milk and milk products (30.4% before and 46.1% after), pulses, seeds, nuts and their products (18.6% before and 49.2% after), spices and condiments (34.0% before and 68.5% after, and vegetables and their products (49.9% before and 76.6% after). Consequently, most of the reported intakes in grams were also influenced across all food groups. Recipes are an important source of food consumption, and their disaggregation should be carefully considered in dietary assessment.

## 1 Introduction

Individuals may report the consumption of single foods, beverages, and recipes during dietary assessment. Recipes, also known as composite dishes or mixed dishes, represent the combination of ingredients from different food sources (1). Depending on the study’s objectives, detailed information from recipes should be gathered during the data collection phase of dietary assessment, which would include collecting details about ingredients and their quantities, the cooking method, the total amount prepared, the number of people served, and the amount of the portion consumed by the participant (or served and leftover). However, in practice, obtaining such detailed information is not always straightforward. Individuals may not recall the details because they were not involved in the recipe preparation or simply do not know or remember. In addition, data collection methods may not have been specifically designed to capture such information (2, 3).

Consequently, during the analysis of dietary survey data, in particular for surveys that did not collect detailed recipe information, it may be necessary to post-disaggregate recipes into ingredients. This step can be crucial for accurately estimating the composition of recipes and their precise contribution to overall food consumption and nutrient intake (4–7). Non-disaggregated recipes can lead to inaccuracies in dietary intake estimates by failing to account for the individual contributions of each ingredient, resulting in either overestimation or underestimation. For example, Sui and colleagues (8) found that disaggregating recipes in the Australian national dietary survey significantly influenced nutrient and food group estimates, resulting in lower intakes of red meat (9%), poultry (25%), and fish (18%) but higher estimates of processed meat (17%). After disaggregation, contributions of foods to total energy (25%), protein (49%), saturated fat (29%), iron (26%), and zinc (38%) were notably higher.

Recipe disaggregation involves identifying all the ingredients and their quantities (and, therefore, proportions) in a recipe. In dietary surveys, this means preparing a food consumption dataset at the ingredient level. Recipe disaggregation also facilitates data harmonization and sharing (8), especially at the international level (9). These are some of the reasons why the European Food Safety Authority (EFSA) has recommended recipe disaggregation in the EU-Menu methodology for national individual dietary studies (10).

Although seen as necessary, recipe disaggregation is not always performed in dietary surveys. There are different reasons for this, including the lack of standardized guidance, recipe source information and the cost-efficiency of applying the procedure during data analysis.

Other non-nutrient dietary indicators may be of interest and will also require recipe disaggregation at the ingredient level to allow proper use and comparability of the data, including assessments focused at the food group level, as well as food safety and environmental evaluations. For instance, the consumption of vegetables may be significantly underestimated, or the classification of foods according to food processing categories may be imprecise if recipes are not broken down into individual ingredients (7, 11, 12). Different studies using the technique of classifying foods into processing categories have highlighted the challenges and limitations imposed by not disaggregating recipes (11, 12). These often arise from the lack of detailed information collected and analyzed during dietary assessments, such as brand names, place of production, and other consumption details. Moreover, more refined analysis can be performed if data are also available at the food item/ingredient level.

International recommendations exist on how to calculate the nutrient values of recipes (13–15). Thus, focusing on the disaggregation of recipes to estimate nutrient intakes, INFOODS (the International Network of Food Data Systems) recommends using the mixed recipe method (15). In this recipe calculation method, the yield factor is applied at the recipe level and nutrient retention factors at the ingredient level, i.e., the retention factor-adjusted nutrient values of the different ingredients are summed and divided by the yield factor for considering the weight change of the recipe during cooking. This method, however, does not express the results for each ingredient, i.e., grams of consumed amounts and nutrient values for each ingredient, which may be required in some dietary analyses. In this sense, the so-called ingredient method may be a possible alternative approach (14).

Additionally, the risk of biased results during recipe disaggregation is non-negligible. To mitigate this risk, using standardized techniques and methodologies for recipe disaggregation can be applied. This includes implementing systematic procedures for ingredient identification and quantification. Given the above, this investigation aimed to assess the influence of recipe disaggregation of a recent national dietary survey in Saint Kitts and Nevis. Furthermore, it seeks to provide recommendations for future assessments and possible guidelines related to best practices in recipe disaggregation.

## 2 Materials and methods

This comparative analysis aimed to evaluate the influence of recipe disaggregation on estimates of food group intakes. A simple and pragmatic recipe disaggregation methodology was specifically developed for this purpose, tailored for the National Individual Food Consumption Survey (NIFCS) conducted in Saint Kitts and Nevis. The analysis focused on differences in frequencies of food group consumption before and after recipe disaggregation.

### 2.1 Study population

The data for the NIFCS in Saint Kitts and Nevis was collected between November 2020 and February 2021. This survey, representative at a national level, utilized a cross-sectional, multi-staged sampling. The Saint Kitts and Nevis Department of National Planning and Statistics Unit provided the number of households and gender distribution sorted by enumeration districts. Households were randomly selected from the enumeration districts. The estimated population of 2021 in Saint Kitts and Nevis was 47,606 individuals (16).

The sample size was calculated to ensure national representativeness and accurate estimations of dietary consumption across geographical regions. We used a 95% confidence level and a 5% margin of error, with a conservative prevalence estimate of 50% due to limited historical data. To accommodate potential non-responses, the sample size was increased by 20%. The three-stage sampling method included a design effect of 1.5, and stratification was based on gender and island. This was achieved with the final sample of 1004 adults, aged 18 to 65 years, from both genders and representative of ethnic groups in the country. Non-citizens, non-residents, individuals outside the indicated age range, bedridden individuals, individuals with chronic disability, and institutionalized persons were excluded from the survey.

The NIFCS was carried out following the guidelines of the Helsinki Declaration and complying with the Saint Kitts and Nevis law on medical research involving human subjects. Ethics approvals to conduct the NIFCS in Saint Kitts and Nevis were obtained from the Campus Research Ethics Committee of the University of West Indies (UWI), Saint Augustine Campus, Trinidad and Tobago, and the Saint Kitts and Nevis Ethics Committee. Permission to conduct the NIFCS was granted by the Ministry of Health, Saint Kitts and Nevis. Informed consent was obtained from all the participants.

### 2.2 Dietary data collection

Twenty-four-hour dietary recalls were collected using computerized tablets equipped with the Caribbean version of myfood24^®^ software, a fully automated online dietary assessment database used to collect and analyze dietary data (17). All databases (food and recipe lists, brandnames, quantification methods, etc.) included into myfood24^®^ were adapted for data collection in the Caribbean (18). A second 24-h dietary recall was applied in a subsample (n = 404), with an interval of three to five weeks from the first collection. The standardized multiple-pass method was used during the interview (19). Furthermore, the 24-h dietary recall data collection covered all days of the week, including weekends. To assist participants in describing the amounts consumed, the Food Portion Quantification Manual developed for Saint Kitts and Nevis was used (20). The fieldwork team, including enumerators and field supervisors, were trained using a virtual platform over five days. They also received a manual with guidelines detailing the survey data collection methods.

The development of the Caribbean version of myfood24^®^ comprised a comprehensive country-specific food list for Saint Kitts and Nevis that contained branded and generic food and drink items, including local foods and recipes. The compiled list of foods and beverages was classified into 16 food groups (cereals and their products; roots, tubers, plantains and their products; pulses, seeds and nuts and their products; milk and milk products; eggs and their products; fish, shellfish and their products; meat and meat products; vegetables and their products; fruits and their products; fats and oils; sweets and sugars; spices and condiments; beverages; food for particular nutritional uses; savory snacks; and composite dishes), as defined by the Food and Agriculture Organization (FAO) (9). The database also contained a recipe builder where users could log ingredients. Additionally, a Caribbean survey-specific composition database comprising 30,412 food items with energy and 46 nutritional components was compiled and standardized. In this database, energy intakes were estimated as follows: Energy intakes (kcal/100 g of an edible portion (EP)) = total protein (g/100 g of EP) × 4 + total fat (g/100 g EP) × 9 + available carbohydrate (g/100 g EP) × 4 + total dietary fiber (g/100 g EP) × 2 + alcohol (g/100 g EP) × 7 (21).

### 2.3 Recipe identification in the survey

Recipes were reported either as single ingredients at the data collection stage (i.e. already disaggregated) or as standard recipes without disaggregation (herein referred to as “non-disaggregated standard recipes”). However, due to resource constraints, the research team opted not to disaggregate all non-disaggregated standard recipes and used a simple and pragmatic methodology to choose which recipes would be further disaggregated. Therefore, only those recipes reported more than three times during the survey or those consumed in large amounts (defined as > = 500 grams per recall) were considered for further disaggregation. In total, seventy-eight non-disaggregated standard recipes were post-disaggregated (21% of 377 recipes) into their consumed ingredient weights. The implicit hypothesis with this decision is that selective disaggregation of frequently reported or large quantity recipes will provide a representative understanding of food consumption without significant loss of validity. Zhang et al.’s study (3) provides empirical support for this decision, indicating minimal loss in validity when recipes were treated as standard in the Dutch National Food Consumption Survey.

### 2.4 Recipe disaggregation procedure

Initially, a mixed recipe calculation method was utilized to assess the nutrient composition of recipes during the planning phase of the 2020–2021 Saint Kitts and Nevis NIFCS. Using the software myfood24^®^, all non-disaggregated composite dishes identified in the system were linked to food composition information.

Building on previous INFOODs (15) work as well as unpublished recipe assessments carried out by the FAO, a procedure of recipe post-disaggregation was developed using the ingredient method to disaggregate the selected seventy-eight non-disaggregated standard recipes. The procedure comprised nine main steps. Briefly, 1) Reported recipes were classified into three groups based on available information: a) Recipes with pre-existing disaggregated information, available from a previous stage of the main survey, measured in a kitchen facility at UWI, b) Adaptable recipes: these were recipes that could be adapted from an existing recipe in the first group, such as switching the type of meat. For example, Curried Chicken and Curried Mutton could be used interchangeably, and c) Recipes that needed to be newly disaggregated due to the lack of previous disaggregation or adaptability of recipes to the categories a and b. 2) Standard recipes were selected through a combination of existing data and consultation with local nutritionists; and 3) Recipes were further classified according to the presence of visible fluid remaining at the end of the preparation. 4) Recipes were disaggregated into ingredient lists. 5) Ingredient measurements were converted into grams. 6) Each ingredient was identified with a code based on the survey food coding system. 7) Edible coefficients, which represent the consumable proportion of each food item, were selected for each ingredient. 8) Yield factors were selected from various sources to account for the loss or gain of moisture (water) and solids (e.g., fat) during these cooking processes. Finally, 9) Calculations were made to determine ingredient consumed amounts and percentages, with adjustments for fluid content in relevant recipes. For more details and references used in each step, see Table 1.

**TABLE 1:** Stepwise procedure used to disaggregate recipes in the Saint Kitts and Nevis Individual Food Consumption National Survey 2020-2021.

#	Step	Description		
1	Classification of reported recipes into three groups based on the type of information available	a) Recipes were classified according to pre-existing disaggregated recipe information, available from a previous stage of the main survey, measured in a kitchen facility at the University of West Indies (UWI). b) Recipes that could be adapted from the first group were identified, for example switching the type of meat e.g. Curried Chicken and Curried Mutton could be interchangeably used. c) recipes that would need to be newly disaggregated due to lack of previous disaggregation or adaptability of recipes to the categories a and b.		
1	Example:	Name of Recipe: Red beans and rice. Classified in the third group (c) since there was no previous recipe recorded in the survey.		
2	Selection of the non-disaggregated standard recipes	Initially, eight recipes had pre-existing disaggregated information available from a previous stage of the survey. These recipes were weighed and prepared from scratch. For the remaining non-disaggregated recipes, a search was done in relevant websites and local recipe books to define the best standard recipe to be used. This decision was confirmed by nutritionists knowledgeable about the local eating habits in the Caribbean.		
2	Example	Standard recipe identified in the website: https://www.africanbites.com/caribbean-rice-and-beans/ Vegetable oil: 1/4 cup; Garlic Cloves: 2-3 units; Onion: 1/2 median unit; Creole seasoning: 2 teaspoons; Long grain Rice: 2 cups; Coconut milk: 13,5 oz can; Red Kidney Beans, drained: 15,5 oz can; Salt: to taste; Pepper: to taste; Chicken broth or water: 2 1/4 cups.		
3	Classification of the identified recipes according to the presence of fluid (with or without)	For the purpose of estimating fluid content separately, only recipes with visible fluid content available/remaining at the end of the preparation were classified as containing fluid. For example: soups and sauces. Dishes such as rice were classified as not containing fluid amounts considering that the water is absorbed by the food and the yield factor takes the fluid into account.		
3	Example	Since water was absorbed in the rice and there was no visible fluid at the end of the preparation, the recipe was classified without fluid.		
4	Disaggregation of the recipe	This step was straightforward, with information coming from the standard recipes identified in Step 2, as stated in the original source. Recipes were disaggregated into the list of ingredients with the description of method of preparation and the household measurements or standard units for all ingredients.		
4	Example	Vegetable oil: 1/4 cup; Garlic Cloves: 2-3 units; Onion: 1/2 median unit; Creole seasoning: 2 teaspoons; Rice: 2 cups; Coconut milk: 13,5 oz can; Red Kidney Beans, drained: 15,5 oz can; Salt: to taste; Pepper: to taste; Water: 2 1/4 cups.		
5	Conversion of household measurements and standard units into grams for all ingredients	For this step, the dietary measurement database built for the survey and embedded into the Caribbean version of myfood24^®^ was consulted (18). If a recipe belonged to the first group that was previously measured in the main survey, its quantities were already defined.		
5	Example	Vegetable oil: 54,4 g; Garlic Cloves, Raw: 11,25 g; Onion, Raw: 60,5 g; Creole seasoning: 2,2 g; Rice, Raw: 444 g; Coconut milk: 382,7 g; Red Kidney Beans: 439,4 g; Salt: 13,85 g; Pepper: 3 g; Water: 540 g.		
6	Identification of the food coding system (i.e. coding of the foods used in the food consumption survey)	This was identified with the so-called European Article Number (EAN) from myfood24^®^ software (18). Foods in this software are identified with their names, without further need of description questions such as facets (e.g. Apple, green, with skin, raw).		
6	Example	Vegetable oil: 20000911010154; Garlic Cloves, Raw: 20000909033575; Onion, Raw: 20000909031506; Creole seasoning: 20000913010205; Rice, Raw: 20000901010038; Coconut milk: 20000903030092; Red Kidney Beans: 20000903010917; Salt: 20000913020120; Pepper: 20000913010174; Water: 20000914020011.		
7	Selection of edible coefficients for all ingredients	The sources of edible coefficients used in the survey to represent the consumable proportion of each ingredient were: West African Food Composition Table (34); FAO/INFOODS Global Food Composition Database for Fish and Shellfish (36); McCance and Widdowson’s composition of foods integrated dataset on the nutrient content of the UK food supply (37), Recipe calculations by the University of West Indies team and assumed values for ingredients (*n* = 3) not found in the aforementioned food composition tables.		
7	Example	The following edible coefficients were applied: Garlic: 0,85 (West African FCT, 2019 – code 04_015); Onion: 0,89 (West African FCT, 2019 – code 04_018). These were multiplied by the original quantities. Garlic: 11,25 g * 0,85 = 9,6 g Onion, Raw: 60,5 g * 0,89 = 53,8 g For the remaining ingredients, edible coefficient was assumed to be 1, and quantities remained the same.		
8	Selection of Yield factors for all ingredients and recipes	The sources for the yield factors to account for the loss or gain of moisture (water) and solids (e.g., fat) during these cooking processes were: West African Food Composition Table (34); Tables on weight yield of food and retention factors of food constituents for the calculation of nutrient composition of cooked foods (dishes) (33); Nutrient Losses and Gains in the preparation of Foods (32); and assumed values for raw ingredients or not located in the tables (*n* = 270).		
8	Example	The following yield factors were applied: Garlic, boiled: 0,95 [Bognar (33), Table 17 “root and tuber vegetables, boiled”]; Onion, boiled: 0,82 (West African FCT, 2019−code 04_112); Rice, boiled: 2,98 (West African FCT, 2019−code 01_134). Garlic, boiled: 9,6 g * 0,95 = 9,1 g Onion, boiled: 53,8 g * 0,82 = 44,2 g Rice, boiled: 444 g * 2,98 = 1323,1 g For the remaining ingredients, yield factors was assumed to be 1, and quantities remained the same.		
9	Calculation of grams and percentages for all ingredients in each recipe.	a. If the recipe was classified without fluid content: the amounts were calculated using the results in step 8. b. If the recipe contained fluids in considerable amounts at the end of the preparation: the amounts were calculated using the results in step 8 AND the fluid content in the recipe was estimated as part of the calculation.		
9	Example	**Ingredient**	**Quantity (g)**	**%**
		Vegetable oil	54,4	1,9
		Garlic, boiled	9,1	0,3
		Onion, boiled	44,2	1,6
		Creole seasoning	2,2	0,1
		Rice, boiled	1323,1	47,1
		Coconut, milk	382,7	13,6
		Red Kidney Beans	439,4	15,6
		Salt	13,9	0,5
		Pepper	3,0	0,1
		Water	540,0	19,2
		Total	2811,9	100

The ingredient weights (in grams) were identified and estimated from the original recipe and then multiplied by the edible coefficient. The result was then multiplied by a selected yield factor. These calculations resulted in the weight of the cooked/consumed ingredient. For the total weight of the recipe, the weight of the cooked/consumed ingredients was summed, and the percentage of each ingredient was estimated. In addition, extra calculations were required for the recipes with water or visible fluid content. These included the cooked recipe weight and the fluid content of each cooked recipe.

### 2.5 Data and statistical analysis

Where available, both days of food consumption were averaged for each individual. The proportion of consumers was expressed in relative frequency, and the food group consumption was expressed in grams by means and confidence intervals (95%). Considering the expected frequency in each category, either the chi-square or Fisher’s exact tests were applied to assess the significance of differences in frequency of food group consumption frequencies before and after disaggregation. A p-value less than 0.05 was considered statistically significant. All analyzes were carried out using R software for statistical computing, version 4.0.2. The R library “survey” was used for model fitting, aiming to include the sample weights (22).

## 3 Results

A total of 440 recipes were reported in the 2020–2021 Saint Kitts and Nevis NIFCS. Of these, 65 recipes were reported with ingredient details during the interview, while 375 were reported with no indication of ingredients used. Seventy-eight non-disaggregated recipes were further identified as recipes to be further disaggregated because they were reported frequently or consumed in larger amounts (See Supplementary material for the list of recipes).

From the 78 recipes, three recipes had the ingredients identified from previously existing information, five were adapted from existing information, and 70 new recipes were identified from relevant recipe books (23, 24) and the internet (25–29). The decisions taken were confirmed by nutritionists knowledgeable about the local eating habits in the Caribbean. While there is always a level of uncertainty about such decisions and in the absence of recognized standard recipes for the country, the best possible recipe was chosen. Concerning the determination of fluid content, four recipes contained visible liquid when ready for consumption at the end of preparation. Consequently, their recipe calculation accounted for the quantification of fluids.

With the standard recipes identified, the ingredients of each recipe were itemized, and amounts were described using household measurements that were converted into grams using the food measurement information used in the survey (e.g., 1 tablespoon of milk is equivalent to 15.6 grams). In addition, the physical state of each item was identified before and after preparation (e.g., raw, cooked, canned). Each recipe ingredient was assigned a food code, which was subsequently used to merge survey files, including composition data. Furthermore, edible coefficients and yield factors were identified, and calculations were performed to determine the proportions of each ingredient.

The 78 disaggregated standard recipes generated information for 326 unique ingredients, which were repeated in some recipes, resulting in 899 rows of ingredients in the recipe database. These ingredients were from the following food groups: vegetables and their products (29.1%), spices and condiments (17.4%), cereals and their products (13.4%), milk and milk products (6.8%), meat and meat products (6.5%), fats and oils (6.3%), pulses, seeds, nuts and their products (5.6%), fruits and their products (3.8%), eggs and their products (3.3%), beverages (2.3%), fish, shellfish and their products (2.3%), sweets and sugars (1.6%), roots, tubers, plantains and their products (0.7%), and a composite dish, which referred to chicken broth (0.7%). After incorporating these ingredients into the main survey dataset, 6284 new food (ingredient) rows were generated. These disaggregated recipes contributed to 15.3 percent of the total energy intake of the population, while the remaining non-disaggregated standard recipes (n = 297) contributed to 12.6 percent.

Overall, there was a significant difference in the estimated diet when comparing disaggregated and non-disaggregated recipes. The analysis revealed that the proportions of consumers across the different food groups increased dramatically for some food groups after recipe disaggregation (Table 2), with the most remarkable difference for fats and oils (6.9% before and 44.5% after), pulses, seeds, nuts and their products (18.6% before and 49.2% after), vegetables and their products (49.9% before and 76.6% after), cereals and their products (81.3% before and 94.7% after), and meat and meat products (59.7% before and 71.4% after). Consequently, the reported consumptions were significantly different across all food groups, except for beverages, foods for particular nutritional uses, fruits and their products, savory snacks, and sweets & sugars. As expected, composite dishes were a group that decreased its proportion of consumers and amounts consumed since part of these dishes/recipes were post-disaggregated (83.8% before and 60.1% after).

**TABLE 2:** Proportion of consumers and mean (confidence interval, CI) consumption (in grams) of foods before and after recipe disaggregation in the National Food Consumption Survey in Saint Kitts and Nevis Survey, 2020–2021.

	Before recipe post-disaggregation			After recipe post-disaggregation			
Food group*	**% consumers**	**Mean (grams)**	**CI**	**% consumers**	**Mean (grams)**	**CI**	***p*-value^†^**
01 Cereals and their products	81.3	137.6	129.0–146.3	94.7	189.3	180.5–198.1	< 0,001
02 Roots, tubers, plantains and their products	26.7	32.2	27.9–36.5	29.4	32.2	27.9–36.5	1,00
03 Pulses, seeds and nuts and their products	18.6	13.5	10.5–16.5	49.2	31.0	27.7–34.5	0,001
04 Milk and milk products	30.4	25.1	21.3–28.9	46.1	32.1	28.2–36.1	< 0,001
05 Eggs and their products	21.7	10.1	8.4–11.8	34.6	13.7	11.8–15.5	< 0,001
06 Fish, shellfish and their products	26.7	43.2	37.0–49.4	38.5	47.5	41.2–53.7	< 0,001
07 Meat and meat products	59.7	61.8	56.9–66.6	71.4	72.3	67.2–77.5	< 0,001
09 Vegetables and their products	49.9	36.9	32.6–41.2	76.6	58.6	53.9–63.4	0,001
10 Fruits and their products	38.2	56.4	49.4–63.5	36.6	56.5	49.5–63.5	0,82
11 Fats and oils	6.9	0.4	0.3–0.5	44.5	3.0	2.6–3.4	< 0,001
12 Sweets and sugars	61.4	37.2	32.8–41.7	62.2	37.4	32.9–41.8	0,71
13 Spices and condiments	34.0	2.4	2.0–2.8	68.5	6.6	5.8–7.3	0,001
14 Beverages	99.8	1102	1068–1136	99.8	1104	1070–1138	1,00
15 Foods for particular nutritional uses	1.7	3.6	1.8–5.5	1.7	3.6	1.8–5.5	1,00
18 Composite dishes	83.8	249.2	234.7–263.7	60.1	124.0	112.7–135.4	0,001
19 Savory snacks	7.9	1.9	1.4–2.4	7.9	1.9	1.4–2.4	1,00

*FAO (9) Not all food groups were reported in the survey.

^†^Chi-square or Fisher’s exact tests were applied to assess the significance of differences in frequencies of food group consumption (%) before and after disaggregation.

Furthermore, this analysis highlighted three crucial aspects: firstly, the uncertainty surrounding the selection of standard recipes and sources in step 2; secondly, the uncertainty involved in selecting edible and yield factors in steps 7 and 8; and finally, the potential conflicts arising from the use of different approaches to disaggregate dietary information within the survey (data not shown).

## 4 Discussion

This study aimed to assess the influence of recipe disaggregation in the 2020–2021 Saint Kitts and Nevis NIFCS, as well as provide recommendations for future dietary assessments. The assessment comprised nine steps, leading to the disaggregation of approximately 21% of the total non-disaggregated standard recipes that lacked ingredient details in the survey. The proportion of consumers and amounts of consumption of the following food groups were influenced by the recipe disaggregation: cereals and their products; composite dishes; eggs and their products; fats and oils; fish, shellfish and their products; meat and meat products; milk and milk products; pulses, seeds, nuts and their products; roots, tubers, plantains and their products; spice and condiments; and vegetables and their products.

The influence of recipe disaggregation on the estimates of food groups has been highlighted by previous research (6, 7). However, the steps and decisions taken for breaking down recipes in nutritional surveys are not always clearly explained. Our work contributes to addressing this gap and highlights three key aspects for further consideration. Firstly, we acknowledge the level of uncertainty associated with the selection of standard recipes and sources utilized. Secondly, we recognize the uncertainty surrounding the selection of edible and yield factors. Lastly, we explore the potential conflicts in using different approaches to disaggregate dietary information within the survey.

Selecting which standard recipes to consider in a post-disaggregation method involves an important degree of uncertainty related to the information sources used and the fact that only one version of each recipe is used for the disaggregation work. In the absence of established, nationally recognized standard recipes, the best possible recipe was chosen based on the information available. Different culinary practices are expected across households, and this may be even more complex if we consider recipes eaten away from home (30, 31). Moreover, depending on the size of the region/country being assessed and the level of variation in recipe preparation in different contexts, the variety of recipe possibilities may increase. The degree to which these factors influence the recipe disaggregation procedure and its conclusions is not well understood. Previous work from Zhang et al. (3) assessing the extent of standard recipe modifications in the Dutch National Food Consumption Survey indicated that there seems to be a minor loss in validity for food group and nutrient intake if no recipe function is available in the data collection software, and recipes are always treated as standard. We believe that further work on this topic is warranted to confirm the results with different populations, especially considering different population/country sizes, cultural habits and frequency of recipe preparation. The influence of using standard recipes in Saint Kitts and Nevis instead of recipe variations may not be large, considering the relatively small population size (less than 50,000 habitants) (16).

The selection of appropriate edible and yield factors also adds a substantial degree of uncertainty in the assessment of recipe disaggregation. Ideally, we would have preferred to use local factors, but these were not available and, therefore, pragmatic choices were made. The lack of factors is even more problematic to determine the recipe total yield factors used in the procedure for recipes with visible fluid. One could argue that the available and often used references for these factors (14, 32–34) are sufficient and enable the application of the recipe disaggregation procedure. Nonetheless, we reinforce the need for improved and culturally-specific yield and edible factors and the need to assess the influence of “universal” factors in assessments within and across countries.

The procedure for recipe disaggregation described in this manuscript focused on enabling a better assessment of food consumption reported in the 2020–2021 Saint Kitts and Nevis NIFCS, using an adapted ingredient method. Nevertheless, this is not the only procedure to disaggregate and calculate recipes and to consider ingredient information in dietary assessments, as at least three procedure types exist (i.e., recipe method, ingredient method, and mixed method) (15). A recipe calculation method had already been considered for assessing the nutrient composition during the planning phase of the NIFCS. Using the myfood24^®^ software, all composite dishes identified in the system were linked to food composition information, and the composition of new recipes was calculated using the mixed recipe method. While the ingredient method applies the yield factor at the ingredient level, the mixed method applies it at the recipe level. The mean of the yield factors of the ingredients may not be the same as the true yield factor of the recipe. Possible inconsistencies in using the two procedures within the same survey (mixed method in the planning phase for some recipes, and ingredient method in the data analysis phase for other recipes) should be further explored. Furthermore, the situation observed in the NIFCS survey may not be the same as for other surveys, where food composition data may be linked only after the dietary data is collected, incorporating nutrient quantification within the recipe disaggregation procedure or in parallel. We anticipate that data analysis capable of integrating both procedures and accommodating various user needs in dietary assessment would be preferable.

Some limitations of this assessment are to be acknowledged. First, not all non-disaggregated standard recipes reported in the survey were disaggregated due to resource constraints. Nonetheless, the disaggregated recipes (*n* = 78) represented 15.3% of the total energy intake of the recalled data, a proportion similar to the remaining non-disaggregated ones (*n* = 297; 12.6% of the total energy intake). Therefore, it is expected that the remaining recipes that have not been disaggregated in the survey would have a similar influence on the estimates of food consumption. The huge amount of additional work might not justify their non-disaggregation. Further evaluations to assess the effects of these decisions or other proposals are, however, needed. Second, the distribution of the food group consumption have not been adjusted for within-person variability. Because different consumption frequencies were observed for the other food groups under assessment, modeling of the variability would require different model types and this imposed technical challenges. Given that, we opted not to adjust the data for within-person variability. Lastly, the food coding used in this assessment was specific to the country. However, to facilitate data harmonization and dissemination, the FoodEx2 system—a standardized tool for classifying and describing food (35)—was applied to the St. Kitts and Nevis national dietary survey data. This data was subsequently shared on the FAO/WHO GIFT platform, where all datasets are coded using FoodEx2. This system automatically assigns individual food items into food groups. Furthermore, FAO has been supporting EFSA in expanding the FoodEx2 catalogue to make it suitable for global use, beyond its initial focus on European countries. As part of this effort, several food items not commonly consumed in Europe were added to the catalogue.

In conclusion, the study conducted as part of the 2020–2021 Saint Kitts and Nevis NIFCS demonstrates the significance of recipe disaggregation in enhancing our understanding of dietary intake. The findings revealed substantial changes in the proportion of consumers across different food groups after recipe disaggregation, notably for fats and oils, pulses, vegetables, cereals, and meat. These adjustments underscore the importance of considering disaggregated recipes in dietary assessments to obtain a more accurate representation of food consumption. In addition, we believe that the method used was a feasible and optimal recipe disaggregation method, which fits the purpose of our assessment. There are, however, some limitations and challenges to be acknowledged, which may be further considered in the future: (1) the definition of standard recipes is not straightforward. There may be many variations of the same recipe within one region and/or country, and the lack of precision in the dietary collection of recipe information imposes uncertainty about the chosen version of the recipes and their disaggregated amounts; (2) there is a lack of country-specific edible and yield factors. Specifically, there is an important gap in existing recipe yield factors, which hampers the quantification of ingredients from recipes with visible fluids; and (3) the use of different approaches to disaggregate and calculate recipes within the same survey may lead to possible inconsistencies, and this should be further explored.

## Data Availability

Publicly available datasets were analyzed in this study. This data can be found here: https://www.fao.org/gift-individual-food-consumption/data/en/.
