# Corrigendum: The influence of recipe disaggregation in dietary assessment: results from the national food consumption survey in Saint Kitts and Nevis

**DOI:** 10.3389/fnut.2025.1629372

**Published:** 2025-06-05

**Authors:** Sandra Patricia Crispim, Vanessa Cardozo Mendes Elias, Latoya Matthew-Duncan, Isabella Francis-Grandeson, Fransen Jean, Victoria Padula de Quadros, Agnieszka Balcerzak, Rita Ferreira de Sousa, Ariel de Moraes Frauches, Claudia Choma Bettega Almeida, Sharon D. Hutchinson, U. Ruth Charrondière, Bridget Anna Holmes

**Affiliations:** ^1^Postgraduate Program in Food and Nutrition, Federal University of Paraná, Curitiba, Paraná, Brazil; ^2^Health Promotion Unit, Ministry of Health, Basseterre, Saint Kitts and Nevis; ^3^Department of Agricultural Economics and Extension, The University of the West Indies, St. Augustine, Trinidad and Tobago; ^4^Food and Agriculture Organization of the United Nations (FAO), Bridgetown, Barbados; ^5^Food and Agriculture Organization of the United Nations (FAO), Rome, Italy; ^6^Formerly of Food and Agriculture Organization of the United Nations (FAO), Rome, Italy

**Keywords:** recipes, food consumption, dietary survey, 24-hour dietary recall, caribbean

In the published article, there was an error in the **Copyright statement**. The statement was incorrectly written as “Copyright © 2024 Crispim, Elias, Matthew-Duncan, Francis-Grandeson, Jean, de Quadros, Balcerzak, de Sousa, Frauches, Almeida, Hutchinson, Charrondière and Holmes. This is an open-access article distributed under the terms of the Creative Commons Attribution License (CC BY). The use, distribution or reproduction in other forums is permitted, provided the original author(s) and the copyright owner(s) are credited and that the original publication in this journal is cited, in accordance with accepted academic practice. No use, distribution or reproduction is permitted which does not comply with these terms.”.

The corrected statement should have been written as “Copyright © 2024 Food and Agriculture Organization of the United Nations. This is an open-access article distributed under the terms of the Creative Commons Attribution License (CC BY). The use, distribution or reproduction in other forums is permitted, provided the original author(s) and the copyright owner(s) are credited and that the original publication in this journal is cited, in accordance with accepted academic practice. No use, distribution or reproduction is permitted which does not comply with these terms.”

In the published article, the **Author Disclaimer** was mistakenly not included in the publication. The missing material appears below:

