# Correction: Differential analysis of the quality and soil microhabitat of *Epimedium koreanum* Nakai under different cultivation methods

**DOI:** 10.3389/fmicb.2025.1634518

**Published:** 2025-06-06

**Authors:** Yonggang Zhang, Huiling Hou, Yanxia Li, Kexin Li, Hongmei Lin

**Affiliations:** ^1^College of Chinese Medicinal Materials, Jilin Agricultural University, Changchun, China; ^2^Cultivation Base of State Key Laboratory for Ecological Restoration and Ecosystem Management of Jilin Province, Ministry of Science and Technology, Changchun, China

**Keywords:** *Epimedium koreanum* Nakai, rhizosphere microbial, pharmacodynamic components, soil properties, soil enzyme activity

In the published article, there were errors in Affiliations.

Instead of

“^1^Cultivation Base of State Key Laboratory for Ecological Restoration and Ecosystem Management of Jilin Province, Ministry of Science and Technology, Changchun, China

^2^College of Chinese Medicinal Materials, Jilin Agricultural University, Changchun, China.”

It is corrected to:

“^1^College of Chinese Medicinal Materials, Jilin Agricultural University, Changchun, China

^2^Cultivation Base of State Key Laboratory for Ecological Restoration and Ecosystem Management of Jilin Province, Ministry of Science and Technology, Changchun, China.”

The authors apologize for this error and state that this does not change the scientific conclusions of the article in any way. The original article has been updated.

